# Principles for Developing Patient Avatars in Precision and Systems Medicine

**DOI:** 10.3389/fgene.2015.00365

**Published:** 2016-01-08

**Authors:** Sherry-Ann Brown

**Affiliations:** Division of Cardiovascular Diseases, Department of Medicine, Mayo ClinicRochester, MN, USA

**Keywords:** precision medicine, systems medicine, SuperModels, DISCRETE, electronic health record, patient avatar, virtual patient, digital patient

## Introduction

**Figure 1:**
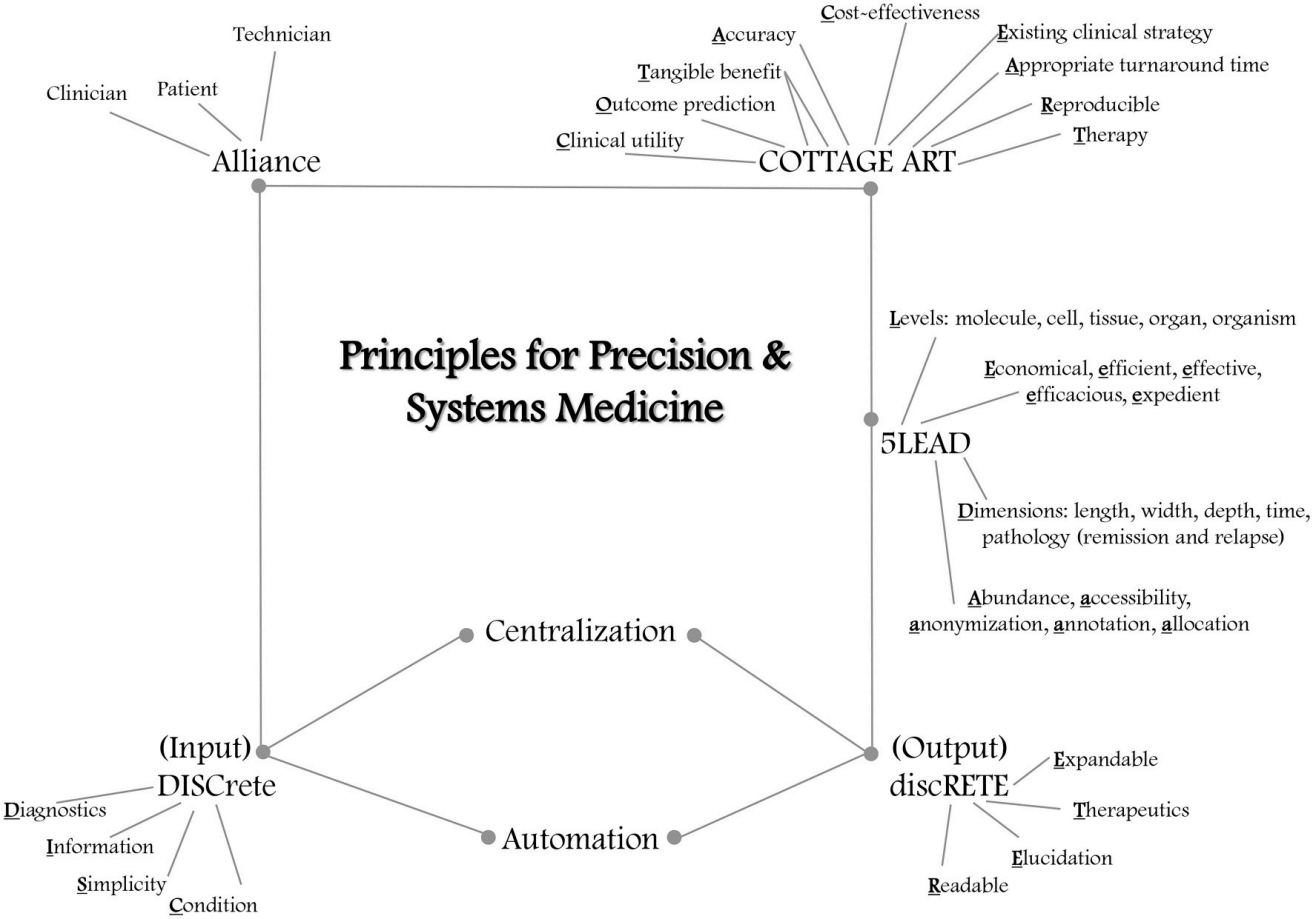
**Emerging principles for precision and systems medicine**. A patient-clinician-technician alliance will be needed to work effectively with patient avatars in precision and systems medicine. The technician will feed data from the clinician and the patient into the SuperModel (in conjunction with systems medicine counseling; Brown, in review), with input that is DISCrete: employing or amenable to diagnostics, providing relevant information, appealing to simplicity using small prototypes specific to a particular condition within the comprehensive SuperModel. Centralized repositories will facilitate model automation. The resulting output will be discRETE: readable, with elucidation of individualized prognosis, recommending therapeutics, and allowing for expandable iteration when new material becomes available. The pentad principles 5LEAD should be used to guide use of SuperModels. Physiological replicas should be prepared at five levels: molecule, cell, tissue, organ, and organism, even if all five levels will not always be simultaneously called out in a DISCRETE. Implementation of SuperModels should be economical, efficient, effective, efficacious, and expedient (5E). Accurate annotation, abundance of replication, and allocation of systems medicine resources, particularly to communities in most need, should ensure anonymization and safe secure accessibility to EHR-integrated data. Patient data is mapped in 5D, regarding length, width, depth, time, and pathology (disease remission and relapse). Finally, the clinical utility, accuracy, tangible benefit, and cost-effectiveness of SuperModel or other precision or systems medicine output recommendations should be demonstrated in reproducible assays, before existing clinical strategy is replaced or complemented; suggested therapy and outcome prediction should be provided by the systems medicine simulations, with appropriate turnaround time (COTTAGE ART).

The day is drawing near when each patient can be made into a SuperModel—not a fashion model, but a digital or virtual model (see companion paper Brown, in review). Such representation of a patient can also be termed a patient avatar (The Discipulus Project, 2013). Principles to help guide successful implementation of these SuperModels are addressed in this article, including efficiency and cost-effectiveness (Figure 1).

## Patient-clinician-technician alliance

A patient-clinician-technician alliance will be needed for optimal care. The patient will interact with the clinician and technician, while the technician guides input of information into the SuperModel. Upon availability of model results, the technician will help interpret output for the clinician and the patient (The Discipulus Project, 2013). Multiple levels of support among the patient's health care team will be needed for shared clinical decision-making, buttressed by patient engagement and involvement of kinship and social networks. Thus, the health care team, patient, and kinship and social networks should be educated about the probabilistic (as opposed to deterministic) nature of SuperModel output. This would be facilitated by systems medicine counseling (Brown, in review). Various clinical decision support (CDS) tools are being developed for research and clinical use (e.g., http://www.myhealthavatar.eu, http://www.myresults.org), which help physicians decipher results and communicate with patients and their networks.

## Centralization and automation

Natural-language processing and cognitive computing have been harnessed to create CDS tools, combining guidelines, recommendations, and primary scientific literature with a virtual advisor trained by medical experts to guide individualized care. One example is the preliminary MD Anderson Oncology Expert Advisor, in collaboration with the International Business Machines (IBM) Corporation's Watson (Savage, 2014). SuperModels should be similarly vetted. To facilitate this, centralized repositories of systems medicine pathways should be widely available to international health care, investigative, and regulatory teams for inclusion in the SuperModels. Models should span various databases such as the Virtual Cell (http://www.vcell.org) and run on worldwide accessible supercomputers such as IBM cores (http://www.ibm.com) or OpenScience grid (http://www.opensciencegrid.org). Semantic interoperability (The Discipulus Project, 2013) should be optimized among various programming, pathway, and ontological languages, e.g., Molecular Interaction Map (MIM) (Tortolina et al., 2015) and SBML (The SBML Team, 2012), in order to combine platforms. With such standardization and homologation, systems medicine technicians will be able to select a subset of relevant model frameworks from a suite of SuperModel prototypes in an automated fashion. Within these frameworks, specific categories will be selected, including area of medicine, nation, and area of the country, with routing to an appropriate regional supergrid. The selection should yield the right model phenotype best suited to address the precision medicine question: What is the right care at the right time for this particular patient? The SuperModel interface would therefore facilitate personalization of appropriate model prototypes. The output would provide customized analyses and recommendations available to the technician and clinician. This information would also be distilled and made available to patients in electronic portals, via desktop or laptop computers, smart phones, and other means of accessing the internet and mobile health (mHealth) applications.

## Discrete

SuperModels data should be queriable in discrete interactive blocks that can be called out to tailor processes to the patient encounter economically and efficiently. Each model package or module is here termed a SuperModule, or a DISCRETE—short for diagnostics, information, simplicity, condition, readable, elucidation, therapeutics, and expandable. The mnemonic DISCRETE encapsulates several guiding principles for precision and systems medicine. Some of these principles elaborate on suggestions made by The Discipulus Project to help guide incorporation of computational models into clinical practice (The Discipulus Project, 2013).

Input to SuperModels to access a DISCRETE should consider diagnostics in a directed fashion. Data from diagnostic procedures would be made available to the SuperModels through integration of electronic health record (EHR), mobile health (mHealth) (Lippman, 2013), telemedicine (McManus et al., 2014), and other platforms, tools, or media. The SuperModel package itself can then be used as a diagnostic tool by pooling data from various sources. Information about patient characteristics can be selected from dropdown menus for risk factors (modifiable and non-modifiable, traditional and non-traditional), genes, environment, lifestyle, behaviors, adherence to treatment recommendations, preferences, socioeconomics, location, and access. Such information would also be pulled in from patient records. The DISCRETE ensures simplicity by providing small modules for input and analysis by the technician. Each module, however, would be comprehensive and specific to a particular condition or disease such as cancer, stroke, or heart disease, each of which often associates with multi-organ contributors, complications, or failure.

Output from a DISCRETE (or SuperModule) should be readable by the technician and by various computer resources, including the patient portal. Module results should lead to elucidation of patient-specific prognosis. Therapeutics should also be recommended. DISCRETEs should be testable, verifiable, and iteratively expandable as new material becomes available in literature or from the patient record. In fact, *in principio* modules can be validated as compact DISCRETEs prior to integration into SuperModels.

## 5LEAD

This section introduces a second novel mnemonic that captures pentad principles for precision medicine, which are in part inspired by assessing the Digital Patient roadmap (The Discipulus Project, 2013).

Pioneering principles for computational avatars and other systems medicine data in precision medicine may be succinctly encapsulated in “5LEAD.” Systems biology tools should simulate physiology at 5 Levels: molecule, cell, tissue, organ, and organism. This allows for analyses that can be fit to available molecular, genomic, histopathological, or other clinical data available at these various spatial and geometric scales among interacting organ systems.

For incorporation into clinical practice, implementation should be economical, efficient, effective, efficacious, and expedient (5E). This is needed to ensure affordability and lasting clinical utility in real-time. Health care is an expensive entity that has so far been reactive to patients' disease. Now we are moving toward proactive, predictive, and preventive care. The Discipulus Project, for example, is funded by the European Commission with a call to provide affordable and personalized primary, secondary, and tertiary preventive care to decrease health care costs (The Discipulus Project, 2013). One might anticipate that other governments, academia, industry, and health insurance companies will serve as partners to stratify and optimize medical care for chronic public health burdens, such as stroke, cancer, and heart conditions. One might expect that collaborative cost-sharing will be eased by decreasing figures for systems medicine data procurement, similar to human genomic sequencing costs (currently ~$1000) down from ~$400 million 10 years ago (Collins and Varmus, 2015). Existing parsimonious methodologies also include phenomenological and mechanistic reduction of complex physiological models (Brown et al., 2011, 2015; Brown and Loew, 2012, 2014), and adaptive semi-supervised recursive tree partitioning (Wang, 2015). Such methods minimize model parameters while maintaining authenticity, and efficiently and effectively retrieve patient similarity data, respectively. SuperModels could conceivably require massive computer power for execution. However, additional steps should be followed to avoid prohibitive computational needs. One critical step would employ Quasar, a new engineering paradigm that increases server efficiency from a typical 20% to an impressive 70% (Delimitrou and Kozyrakis, 2014).

Abundance, accessibility, anonymization, annotation, and allocation (5A) are also needed. Accurate annotation and broad replication (abundance) of systems medicine resources should occur across nations. Allocation to communities in most clinical need should be enhanced to provide safe, private (anonymization), and secure access to SuperModels. At-risk, understudied, or underrepresented populations should be oversampled to contribute to universal avatars. These can be used as starting points for diverse patient consultation on the way to personalization.

Precision and systems medicine will occur in five dimensions (5D). Each patient's biometrics will be closely matched holographically regarding length, width, depth, time, and pathology (disease remission and relapse). Long-term prediction, particularly for chronic diseases which pose an economic health care burden, will be key for management of customized care and resources (The Discipulus Project, 2013).

## Cottage art

A third novel mnemonic calls upon important additional principles suggested by other groups. In order to compose the mnemonic, one may consider that in the modern theme park of science, systems biology (the study of whole organisms through life and natural sciences) (Likić et al., 2010), biomarker discovery (Ge and Wang, 2012), and pharmacogenomics (response to drugs based on genetics) (Wang et al., 2011) sit next to bench-to-bedside -and-clinic translation (Ritchie, 2012). There, each discipline together takes a ride to help decide how numeric quantitation in the human body can help with understanding the expression, as well as concentration and regulation of normal protein and also mutation. There, results of model studies with mice used as examples can be compared with corresponding phenotypes from multinational human samples. This is spurred on by the hope that methodology integration can help with pathophysiology elucidation (Milward et al., 2012; Botling et al., 2013), with the ultimate goal of human disease mitigation.

Yet, before any biotechnological advance in precision medicine can become ubiquitous, certain principles are needed to be efficacious (Pereira and Weinshilboum, 2009). The testing done for each patient must have clinical utility. Before replacement of existing clinical strategy, the test should demonstrate superiority. There ought to be tangible benefit and cost-effectiveness that place the patient's needs before the test. Biotechnology in precision medicine testing must allow for outcome prediction, with appropriate turnaround time facilitation. All of these requirements can be put in place with ease of memory, using a mnemonic: COT ACE.

Precision Medicine in clinical practice can go hand in hand with risk stratification and biomarker discovery in a way that augments accuracy (Kullo and Cooper, 2010). Of course, this should involve a reproducible assay, with anticipation of available intervention via therapy. This interaction of science with the clinical, in part, may also be governed by the mnemonic: ART.

If we put the two mnemonics together, we generate: COTACE ART. Given the liberty we take with mnemonics and names of clinical trials, we could choose to remain in denial, believing that C is equivalent to G, and change the mnemonic to: COTTAGE ART. Many mnemonics will come and go as will clinicians and researchers, but principles stand the test of time. For this reason COTTAGE ART may persist in our minds and favorably furnish a useful guide. Perhaps as part of the Precision Medicine Initiative (http://www.nih.gov/precisionmedicine), COTTAGE ART can modify how clinical medicine has been practiced and enhance the landscape that has been established.

## Looking ahead

Already computational avatars have associated with improvement of health outcomes (The Discipulus Project, 2013). Clinical trials to validate and demonstrate efficacy of computational avatars and SuperModels as decision aids will continuously be needed to guide clinical implementation, initially in pilot studies with ~200–500 patients, then with larger cohorts such as 10,000 patients (or 1 million, as in the proposed precision medicine initiative cohort; Collins and Varmus, 2015). Among other capabilities, Watson uses cognitive computing to simplify and expedite matching of patients with suitable clinical trials simultaneously underway around the globe (Lewotsky, 2014). Public–private partnerships will likely be forged by the Food and Drug Administration (FDA) to determine regulatory processes to help guide clinical translation of genomic testing (Evans et al., 2015) and other systems medicine data. The use of these technologies in clinical practice to individualize patient care should be guided by these principles that can be well-remembered with the three mnemonics presented in this paper.

Important too is optimizing patient engagement. This is already being done by providing access to avatars through electronic portals, and facilitating communication between patients and their health care teams (e.g., http://www.myhealthavatar.eu, http://www.myresults.org). Open avatar communities should be created to allow for catalytic patient-patient peer interactions to improve personalized self-care. This will take “peer-to-peer healthcare” observed on social media platforms (Fox, 2011) even further and synch this behavior with the best information possible for the particular patient in the form of an avatar. These initiatives can be merged through common clinical user interfaces for use on computers of various shapes and sizes, including smart watches and glasses (The Discipulus Project, 2013).

In conclusion, a myriad of principles have been suggested to help guide the use of interdisciplinary models, consortia, and platforms in clinical practice. In the context of these principles, emerging comprehensive multidimensional computational avatars integrated with wearable sensors, mHealth technology, telemedicine, and the EHR (i.e., SuperModels) may help optimize patient care in precision and systems medicine.

## Author contributions

SB conceived of, analyzed, designed, drafted, critically revised, approved, and agreed to be accountable for this submitted work.

### Conflict of interest statement

The author declares that the research was conducted in the absence of any commercial or financial relationships that could be construed as a potential conflict of interest.
